# A hypothesis connecting dysgeusia due to defects in ATP-P2X3 signaling and fatigue in myalgic encephalomyelitis/chronic fatigue syndrome: lessons learned from long-COVID

**DOI:** 10.3389/fmed.2026.1808646

**Published:** 2026-04-08

**Authors:** Mythily Srinivasan, Paule Valery Joseph

**Affiliations:** 1 Department of Oral Pathology, Medicine and Radiology, Indiana University School of Dentistry, Indianapolis, IN, United States; 2 National Institute on Alcohol Abuse and Alcoholism, National Institutes of Health, Bethesda, MD, United States; 3 National Smell and Taste Center, National Institute on Deafness and Other Communication Disorders, National Institutes of Health, Bethesda, MD, United States

**Keywords:** adenosine triphosphate, bioenergy deficit, long-COVID, ME/CFS, taste dysfunction

## Abstract

Myalgic encephalomyelitis (ME)/chronic fatigue syndrome (CFS) is a neuroimmune disease characterized by debilitating post-exertional malaise (PEM), brain-fog/cognitive problems, and dysregulation of the autonomic nervous system. Currently, there are no objective biomarkers for ME/CFS despite decades of research. Here, we compile evidence from literature that supports taste dysfunction, particularly alterations of taste perception mediated by Type II taste receptor cells, may be a critical underrecognized feature of ME/CFS. The impetus is drawn from the emerging evidence of clinicopathological similarities between long-COVID and ME/CFS. We discuss in parallel the mechanisms of cellular metabolism, inflammation, vascular dysfunction, and autonomic dysregulation in ME/CFS and long-COVID pathophysiology. We postulate that mechanistically, dysregulation of ATP signaling through P2X2/P2X3 purinergic receptors underlies both gustatory impairment and core ME/CFS symptoms. Adopting information from the NIH-RECOVER shared resources, we present evidence that suggests chemosensory dysfunction as a potential indicator of progression/severity of PEM. We discuss standardized taste testing as a non-invasive screening tool complementary to molecular biomarkers for ME/CFS. Notwithstanding, we acknowledge the limitations, confounding and contributing factors such as medications and deficiencies that may exacerbate or independently cause taste-related symptoms in ME/CFS. In conclusion, we present a compelling case for the multi-factorial role of taste dysfunction in ME/CFS and suggest specific research priorities for investigating the relationship between chemosensory function and post-viral chronic illness.

## 1 Introduction

Myalgic encephalomyelitis/chronic fatigue syndrome (ME/CFS) is a debilitating disease characterized by persistent or recurring symptoms including fatigue, post-exertional malaise (PEM), myalgia, joint pain, sleep disturbances, and cognitive dysfunction (1, 2). Proposed triggers include new or reactivation of latent viral infections, physiological or psychological stressors, and genetic susceptibility (3). Significantly, as the long-term effects of the recent severe acute respiratory syndrome (SARS) coronavirus-2 (CoV2) pandemic unfold, emerging data suggest that post-acute SARS-CoV2 symptom complex (PASC) shares many features of ME/CFS symptoms, including debilitating fatigue, PEM, cognitive issues, and sleep disturbances (3–5). Data from NIH’s RECOVER initiative reveal that new ME/CFS cases are 15 times more frequent than before pandemic and that the increased risk is independent of severity of CoV2 infection or reinfection status (5, 6). Indeed, PEM is recommended as an outcome measure for PASC or long-COVID clinical trials (7).

Yet, an interesting discrepancy is the frequent occurrence of dysgeusia in long-COVID and its paucity in ME/CFS reports (3, 4). Persistent taste dysfunction extending for periods longer than one-year post-CoV2 infection has been consistently reported as a component of PASC (8, 9). Isolated dysgeusia, independent of smell loss, occurred in 5–10% of long-COVID cases (10). Furthermore, longitudinal electronic health records of PASC showed that even in children and adolescents CoV2 reinfection increased the risk of chemosensory dysfunction with an odds ratio of 2.83 (1.41–5.67) (11). Interestingly, an observational study reported that dysgeusia occurred in 19% of cases of long-COVID patients diagnosed with ME/CFS (12). Yet, in general, taste dysfunction has not been recognized amongst the symptom clusters of ME/CFS. This could be due to under-recognition, rather than true absence. Indeed the 2021 guidelines by the National Institute of Health and Care Excellence includes altered taste and smell sensitivities as associated symptoms of ME/CFS (1). Evidence supporting under-recognition has been overlooked. A large Belgian study of 2,073 CFS patients reported that 38–42.4% of patients meeting Fukuda or Holemes criteria experienced altered taste, smell, or hearing (1). Further, existing data are derived from patient self-report, which underestimates true symptom prevalence. This trajectory parallels the historical pattern of other ME/CFS symptoms that were initially overlooked but subsequently validated through objective measurements.

The ME/CFS patient community has long advocated for objective diagnostic biomarkers to validate illness and improve care. In this perspective, we present a mechanistic model highlighting the critical roles of taste dysfunction in ME/CFS with supporting evidence from long-COVID as a natural experiment. In the following sections we first provide a brief overview of physiology of taste and introduce a framework for understanding the shared biological pathways between ME/CFS and long-COVID. We next review evidence linking taste dysfunction and ME/CFS emphasizing adenosine triphosphate (ATP) dependent mechanisms of bioenergy deficit and inflammation. We then provide evidence from a publicly available long-COVID data set that supports taste dysfunction as biomarker for ME/CFS. In conclusion, we propose an ATP-dysfunction based model that integrates these separate but related fields, namely chemosensory dysfunction, in particular taste dysfunction, and ME/CFS.

## 2 The sense of taste and taste dysfunction

Gustation, the sense of taste, is mediated by taste buds located chiefly on the tongue’s papillae. Each taste bud contains 50–100 taste receptor cells (TRCs), classified as Type I supporting cells, Type II cells that detect sweet, bitter, and umami tastes, and Type III cells responsible for sour perception (13). TRCs exhibit a turnover rate of 8–10 days, with loss modulated by apoptosis and extrusion and regeneration mediated by proliferation of stem cells (13). Taste disorders include dysgeusia, hypogeusia, phantogeusia, and ageusia. Age associated increased apoptosis or extrusion of TRCs, genetic variants in bitter TRCs, mutations in ATP-channel proteins, and systemic or local inflammation that impairs TRC regeneration are mechanisms attributed to taste dysfunction (13–15).

Several reports show that patients with long-COVID experience dysgeusia characterized by prolonged metallic or bitter taste in the mouth (e.g., coffee tastes metallic and that chocolate has lost its typical taste) (9, 16). Type-II TRCs that mediate such taste dysfunctions rely on ATP as their primary neurotransmitter. Mechanistically, the tastants bind G protein coupled receptors on type-II TRC’s, activate phospholipase C beta 2 (PLCβ2), generate inositol 1,4,5-trisphosphate (IP3), and release intracellular Ca^2+^, which drives ATP efflux through calcium homeostasis modulator 1 (CALHM1) channels. Subsequently, released ATP activates specific purinergic receptors such as P2X2 and P2X3, on cranial nerves VII, IX, and X, triggering action potentials that are transmitted to central gustatory regions culminating in taste perception (13, 17).

## 3 Similarities in pathophysiological mechanisms of ME/CFS and long-COVID

**Figure 1:**
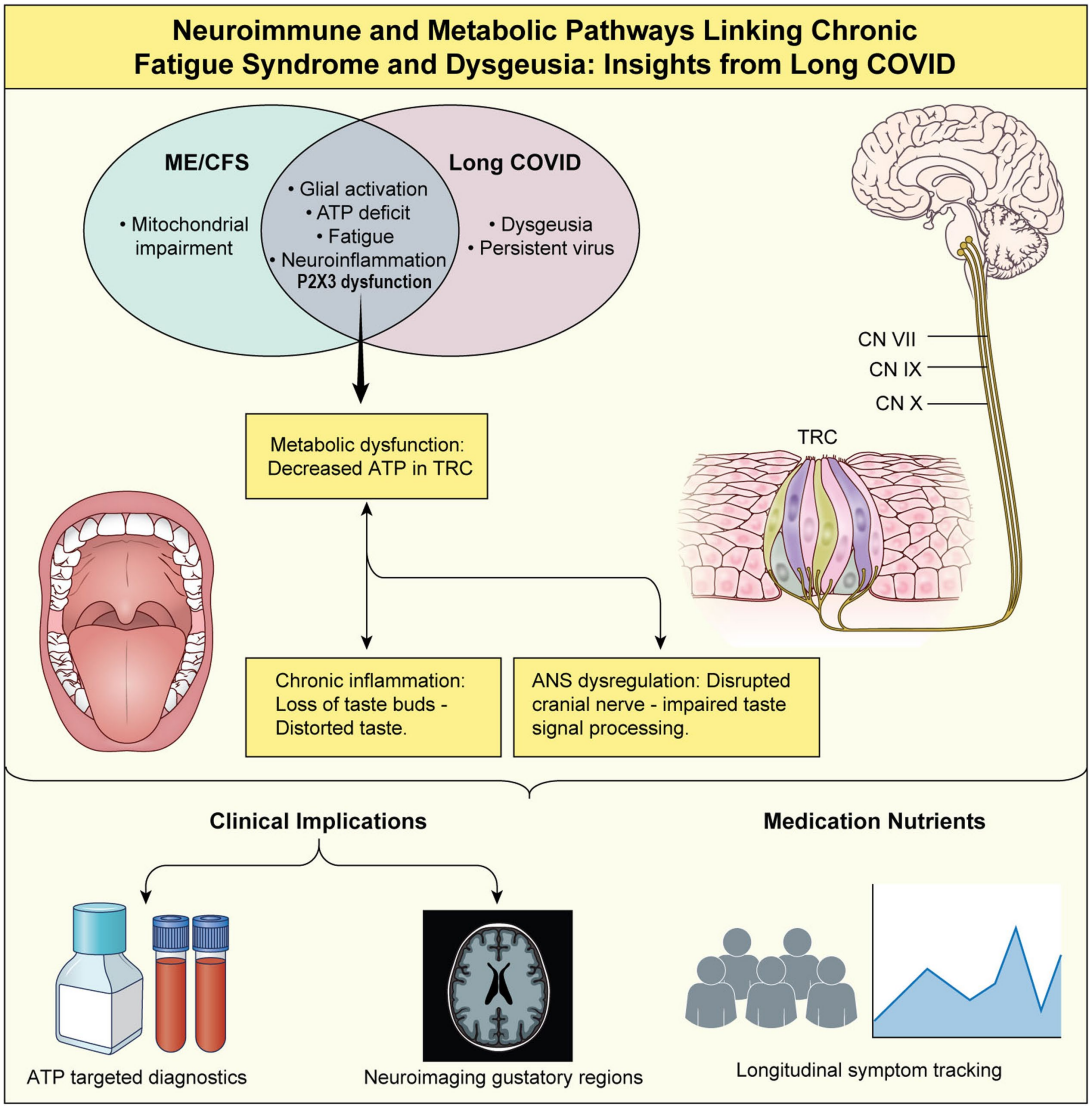
Neuroimmune and metabolic pathways linking chronic fatigue syndrome to dysgeusia: insights from long COVID.

The clinical overlap between ME/CFS and long-COVID has become increasingly apparent as the long-term effects of SARS-CoV-2 infection continue to unfold (4, 5). Similarly, emerging data show that multiple interconnected biological pathways implicated in the ME/CFS pathology are also observed in long-COVID pathology (3, 4). Building on these shared mechanisms, we discuss how dysgeusia, specifically the most frequently reported bitter taste dysfunction, could connect to ME/CFS through energy-dependent mechanisms (Figure 1).

### 3.1 Metabolic-neurotransmitter pathway linking ME/CFS and taste dysfunction

#### 3.1.1 Metabolic dysfunction in ME/CFS

A central feature of ME/CFS is a profound disturbance in cellular energy metabolism due to mitochondrial abnormalities, resulting in low ATP levels. The chronic bioenergy deficit creates a perpetual “anticipation error” for the brain to challenges it cannot meet, manifesting as cognitive impairment, and PEM as a preemptive measure. Thus, a self-perpetuating, pathological feedback loop ensues where the physical energy crisis fuels a neurobiological state of perpetual exhaustion that is not resolved by rest and provides a compelling explanation for the core symptoms of ME/CFS (18, 19). Alternatively, ME/CFS represents a “cell danger response” driven largely by extracellular ATP acting on purinergic (P2X) receptors (20). Activation of multiple P2X receptors including P2X2/P2X3/P2X4 and P2X7 has been associated with immune, metabolic, and neurological dysfunction in ME/CFS. Pertinently, moderate exercise induced significant upregulation of P2X4 and P2X5 expressions in peripheral blood leukocytes of ME/CFS patients and correlated with the severity of PEM (18, 19).

#### 3.1.2 Metabolic dysfunction in long-COVID

Emerging data show that mitochondrial bioenergetic dysfunction is strongly associated with the debilitating symptoms in long-COVID (4, 6). Cellular studies showed that SARS-CoV-2 targets host-cell mitochondria causing structural damage and compromises their function. Consistently, COVID-19 tongue tissues exhibited neurite loss and distorted taste buds (21). Further, several mitochondrial proteins affecting many metabolic pathways were observed to be differentially regulated in peripheral blood mononuclear cells of long-COVID patients (22). Impaired oxidative phosphorylation observed in skeletal muscles also suggests compromised energy-generating capacity in long-COVID. Additionally, blood and skeletal muscle metabolomes of long-COVID patients exhibited higher glycolytic and lower tricarboxylic acid cycle metabolites supporting reduced mitochondrial function (18, 23).

#### 3.1.3 Linking ME/CFS and taste dysfunction

The bioenergy deficits and extracellular ATP dynamics shared between CFS, and long-COVID offer a biologically coherent explanation for why taste dysfunction may both mirror and contribute to ME/CFS pathophysiology. Low ATP levels in ME/CFS could directly impair TRC function by interfering with signal transduction, and neurotransmitter synthesis, thereby disrupting the flow of taste information. Altered purinergic receptor activation patterns could further disrupt taste signaling (17, 24). Mutations in CALHM1, an ion channel gene critical for ATP release by TRCs and hence, taste perception as discussed above, have been linked to cognitive impairment (24). Knockdown of ATP-activated purinergic receptors (P2X2, P2X3) resulted in complete loss of taste (17, 25). In addition, the widespread distribution of P2X and bitter taste receptor genes in gustatory and non-gustatory tissues further substantiates the premise of how perturbations in ATP-mediated taste pathways could contribute to the multisystem dysfunction, characteristic of ME/CFS (17, 26). We extend this framework to suggest that dysgeusia mediated by dysfunction of the highly ATP-sensitive P2X3 receptor could provide a measurable peripheral marker for the systemic purinergic dysregulation in ME/CFS.

### 3.2 Immune dysregulation and inflammation

#### 3.2.1 Neuroinflammation in ME/CFS

A proinflammatory state triggered by infection or autoimmunity (anti-neuronal and anti-endothelial antibodies) underlies the “flu-like” malaise in CFS (4, 6). Further, sustained inflammation activates glial cells, leading to neuroinflammation and hypersensitivity of the hypothalamic paraventricular nucleus, which drives PEM and relapses. Indeed, serum cytokine profiles have been linked to symptom severity in ME/CFS (27).

#### 3.2.2 Chronic inflammation and long-COVID

Emerging data suggest that dysregulated immune responses mediated by persistent SARS-CoV-2 RNA and proteins in various tissues lead to severe fatigue, PEM and cognitive dysfunction in long-COVID (4, 5). The CoV2 damaged host mitochondria act as damage-associated molecular patterns, further amplifying inflammatory responses and worsening fatigue symptoms.

#### 3.2.3 Chronic inflammation strengthens the link between dysgeusia and ME/CFS

Inflammatory conditions in humans have been shown to exhibit a three-fold increase in dysgeusia risk, albeit with high heterogeneity (28). Interestingly, in addition to systemic inflammation, CFS patients also frequently report oropharyngeal lesions like non-exudative pharyngitis and aphthous ulcers (1, 4). Sustained inflammation has been shown to reduce TRC proliferation and impair taste sensation (28). Further, alteration of the P2X3 receptor function by the inflammatory cytokines IL-1β, TNF-α, and IL-6 could modulate ATP signaling in taste cells and sensory neurons (19). This bidirectional relationship between ATP signaling and inflammation creates a positive feedback loop that could sustain both taste dysfunction and broader ME/CFS symptoms.

### 3.3 Vascular dysfunction

Developing evidence suggests that both ME/CFS and long-COVID exhibit vascular dysfunction. In CFS, fatigue severity is correlated with endothelial dysfunction and reduced cerebral and muscular perfusion (29, 30). Fibrinolysis resistant fibrin amyloid microclots that limit oxygen delivery to tissues have been reported in long-COVID. Chronic tissue hypoxia could impair the high-energy demands of TRC-II signaling, as ATP synthesis depends critically on adequate oxygen supply (18, 19). This vascular mechanism may contribute to taste dysfunction, independently or synergistically with the purinergic signaling disruption central to our hypothesis.

### 3.4 Autonomic nervous system dysregulation

Orthostatic intolerance and a blunted heart rate variability observed in ME/CFS are attributed to autonomic nervous system (ANS) dysfunction (2). Disruption of the neural circuitry of the hypothalamus and limbic system within the ANS could interfere with central processing of taste signals, regardless of the health of peripheral taste buds (31). Additionally, a subgroup of ME/CFS patients exhibit autoantibodies against β2-adrenergic and M3 muscarinic receptors, implicating autoimmune disruption of the ANS and salivary glands, both of which are essential for normal taste perception (4, 18). Dysfunction of P2X3 receptors densely expressed in autonomic ganglia and sensory nerve terminals throughout gastrointestinal pathways could lead to autonomic instability and taste disturbance (17). Further, vagus nerve carrying both autonomic efferent and taste afferents from the posterior tongue provides a direct anatomical bridge linking gustatory signaling to autonomic control, reinforcing the biological plausibility of a shared P2X3-mediated mechanism (17, 25).

### 3.5 Other factors influencing taste perception with potential relevance to ME/CFS

#### 3.5.1 Sex-specific considerations

CFS disproportionately affects women, parallelling the well-documented sex differences in taste perception (1, 5). Women tend to be more sensitive to bitter taste and experience fluctuations in their taste sensitivity across the menstrual cycle. The expression of estrogen receptors in TRCs suggest that the hormone estradiol can affect TRC turnover and influence taste perception and could explain higher preponderance of women in ME/CFS (26).

#### 3.5.2 Nutritional deficiencies

Malnutrition and reduced appetite are well-known causes of taste dysfunction and can exacerbate metabolic and neurological disorders. In CFS, gastrointestinal symptoms often drive restrictive eating, leading to vitamin B12 and zinc deficiencies, micronutrients essential for TRC renewal (1, 26). Thus, taste changes may arise from both primary ME/CFS-related alterations and secondary nutritional disturbances, while dysgeusia can worsen nutrient intake and compound the underlying energy deficits.

#### 3.5.3 Medications

Common CFS medications, including tricyclic antidepressants like amitriptyline, can influence taste-receptor activity, suggesting that dysgeusia could be an adverse effect (1).

#### 3.5.4 Potential confounding factors for taste dysfunction as a symptom of ME/CFS

The unique gustatory and olfactory neural tropism of SARS-CoV2 with potential central and/or peripheral effects could contribute to the altered taste perception in long-COVID. Hence, dysgeusia as contributing factor could be restricted to the ME/CFS cohort secondary to CoV2 infection (21, 32). Several additional factors can be etiologically related to gustatory disorders. These include multiple classes of drugs, such as anti-infectives, anti-inflammatory anti-pyretic, antihistamines, antihypertensives, sympathomimetics, anti-diabetics and psychopharmacologic agents (33). Furthermore, oral health issues such as candidiasis and xerostomia (dry mouth) secondary to medications can distort taste sensations. Systemic co-morbidities such as fibromyalgia and Sjogren’s syndrome associated with ME/CFS can also contribute to the taste dysfunction independently or due to medications (14, 34).

## 4 Taste sensitivity as biomarker: evidence from long-COVID

**Figure 2:**
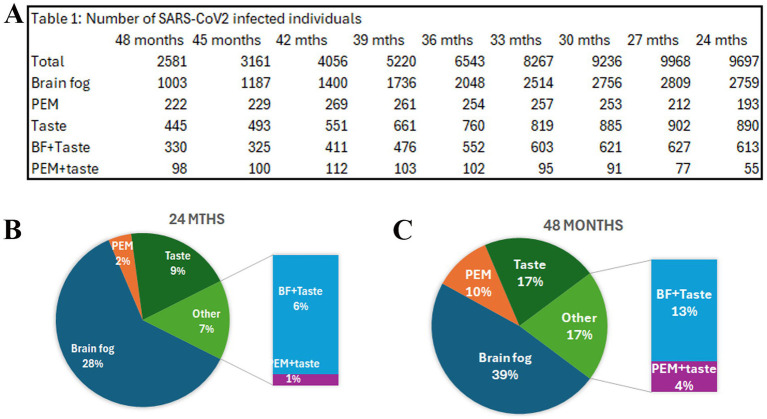
Post exertional malaise (PEM), brain fog and dysgeusia in long-COVID.

Researching COVID to enhance recovery (RECOVER) is a NIH initiated program to understand, diagnose, prevent, and treat long-COVID. Data from this initiative showed that 4.5% of post-COVID-19 participants met ME/CFS diagnostic criteria, compared to 0.6% of uninfected participants (35). As of January 2026, this dataset curated for adult COVID included 84,172 and 58,320 entries from SARS-CoV2 infected and uninfected participants, respectively. We queried the data set of infected participants for the following symptoms over a period of 2–4 years post-index date; (Q1) loss of or change in smell or taste, (Q2) How much does your post-exertional malaise bother you? and (Q3) How much do your problems thinking or concentrating (“brain fog”) bother you?. The percentage of participants reporting “yes, I still have it” for Q1 exhibited an increasing trend, from 9% at 24 months to 17% at 48th month post-index period. Similarly, the percentage of participants responding “very much” for Q2 and Q3 increased with time from 24 to 48 months, consistent with previous reports (Figure 2A). Interestingly, the number of people reporting chemosensory disturbance and PEM increased from 1% in 24 months to 4% in 48 months (Figures 2B, C). Furthermore, the number of people reporting chemosensory disturbance and brain fog doubled from 6% in 24 months to 13% in 48 months. Collectively, these observations suggest that chemosensory dysfunction could represent a biomarker for increased PEM and brain fog, characteristic features of ME/CFS (Figure 2B). However, correlational occurrences are interpreted with caution. The data set does not allow tracking of individual responses, and the combined symptoms could potentially indicate new onset of either symptom. Infrequent reports of taste dysfunction in pre-COVID era in ME/CFS further adds to the cautious interpretation.

Yet, considering that millions of people were exposed to SARS-CoV2, and the increasing trajectory of new ME/CFS, the value of a non-invasive, easily measurable clinical marker such as quantitative taste-testing is very high, even if it is restricted for a subset of ME/CFS patients. Pertinently, ME/CFS therapeutic strategies under development such as P2X3 receptor antagonists and interventions aimed at boosting cellular energy are closely connected to taste perception (18, 19). Yet, with respect to long-COVID data the following limitations are acknowledged: (1) combined documentation of taste and smell changes in the NIH shared data resource and (2) inherent neurotropism of SARS-CoV2 that contributes to the increased chemosensory dysfunction in long-COVID, as opposed to other ME/CFS associated viruses.

## 5 Summary and future directions

Available literature presents substantial indirect and supportive evidence for taste dysfunction as a biomarker or indicator of disease progression or severity of ME/CFS. Future longitudinal studies that systematically track the onset, severity, and fluctuation of taste dysfunction alongside other symptoms in a well-characterized cohort of ME/CFS patients (e.g., Canadian Consensus Criteria or the Institute of Medicine criteria) will determine the significance of dysgeusia, if any, in ME/CFS. As opposed to the emerging screening platforms such as 3D genomics or transcriptomics that need specialized infrastructure, taste testing provides a rapid, inexpensive, non-invasive option that can be repeated frequently in clinical or home settings (36). Quantifying taste preference and thresholds for the five basic tastes and serial assessments can potentially anticipate PEM episodes, offer an objective index of disease progression, and identify individuals needing confirmatory molecular testing. The metabolic-neurotransmitter hypothesis could be addressed by quantifying ATP levels and mitochondrial function in TRCs and using advanced brain imaging to investigate the integrity of gustatory brain regions.

## Data Availability

The original contributions presented in the study are included in the article/supplementary material, further inquiries can be directed to the corresponding author.
